# Incident Cardiovascular Disease Among Adults With Cancer: A Population-Based Cohort Study

**DOI:** 10.1016/j.jaccao.2022.01.100

**Published:** 2022-03-15

**Authors:** D. Ian Paterson, Natasha Wiebe, Winson Y. Cheung, John R. Mackey, Edith Pituskin, Anthony Reiman, Marcello Tonelli

**Affiliations:** a Department of Medicine, University of Alberta, Edmonton, Alberta, Canada; b Department of Oncology, University of Calgary, Calgary, Alberta, Canada; c Department of Oncology, University of Alberta, Edmonton, Alberta, Canada; d Faculty of Nursing, University of Alberta, Edmonton, Alberta, Canada; e Department of Medicine, Dalhousie University, Halifax, Nova Scotia, Canada; f Department of Medicine, University of Calgary, Calgary, Alberta, Canada

**Keywords:** cancer survivorship, coronary artery disease, epidemiology, heart failure, AMI, acute myocardial infarction, CV, cardiovascular, MI, myocardial infarction, SEER, Surveillance, Epidemiology, and End Results

## Abstract

**Background:**

Patients with cancer and cancer survivors are at increased risk for incident heart failure, but there are conflicting data on the long-term risk for other cardiovascular events and how such risk may vary by cancer site.

**Objectives:**

The aim of this study was to determine the impact of a new cancer diagnosis on the risk for fatal and nonfatal cardiovascular events.

**Methods:**

Using administrative health care databases, a population-based retrospective cohort study was conducted among 4,519,243 adults residing in Alberta, Canada, from April 2007 to December 2018. Participants with new cancer diagnoses during the study period were compared with those without cancer with respect to risk for subsequent cardiovascular events (cardiovascular mortality, myocardial infarction, stroke, heart failure, and pulmonary embolism) using time-to-event survival models after adjusting for sociodemographic data and comorbidities.

**Results:**

A total of 224,016 participants with new cancer diagnoses were identified, as well as 73,360 cardiovascular deaths and 470,481 nonfatal cardiovascular events during a median follow-up period of 11.8 years. After adjustment, participants with cancer had HRs of 1.33 (95% CI: 1.29-1.37) for cardiovascular mortality, 1.01 (95% CI: 0.97-1.05) for myocardial infarction, 1.44 (95% CI: 1.41-1.47) for stroke, 1.62 (95% CI: 1.59-1.65) for heart failure, and 3.43 (95% CI: 3.37-3.50) for pulmonary embolism, compared with participants without cancer. Cardiovascular risk was highest for patients with genitourinary, gastrointestinal, thoracic, nervous system and hematologic malignancies.

**Conclusions:**

A new cancer diagnosis is independently associated with a significantly increased risk for cardiovascular death and nonfatal morbidity regardless of cancer site. These findings highlight the need for a collaborative approach to health care for patients with cancer and cancer survivors.

Advances in early diagnosis and treatment have substantially improved clinical outcomes for most patients with cancer in the past 2 decades. As the life expectancy of cancer survivors has increased, so has the likelihood of developing other illnesses after cancer diagnosis. Cancer and cardiovascular (CV) disease share many risk factors, including smoking, lower socioeconomic status, and obesity.^1^ This suggests that patients with cancer constitute a high-risk population for CV disease. Furthermore, many cancer therapies, including chest irradiation as well as systemic therapies such as chemotherapy, are associated with incident CV disease during treatment and in survivorship. It has been hypothesized that the excess CV morbidity of cancer treatments is mediated by direct myocardial and/or vascular injury as well as indirectly through adverse effects on lifestyle behaviors.^2^

However, recent population studies have yielded conflicting results on the long-term CV risk of cancer survivors. One study using data from the Surveillance, Epidemiology, and End Results (SEER) program demonstrated a higher risk for CV death among 234,256 cancer survivors compared with the U.S. general population, particularly in the first year after cancer diagnosis.^3^ However, another SEER-based study showed that cardiac mortality in a cohort of 347,476 patients with breast cancer was not increased compared with age-matched control subjects during long-term follow-up.^4^ Although these studies considered the effects of age, ethnicity, and cancer-related variables, they did not adjust for other risk modifiers, including prior CV disease, hypertension, diabetes mellitus, and dyslipidemia. Indeed, administrative databases confirm a high prevalence of CV disease in patients with cancer relative to cancer-free control subjects.^5^ Prior work on CV risk in cancer survivors has also been limited in scope and has typically included study of only 1 cancer type and 1 CV outcome.

Access to multiple health data repositories in Alberta, Canada, permits the construction of well-characterized, population-based cohorts with extensive profiling of cancer and CV disease. Therefore, we designed this study to investigate the risk for incident CV events among people with histories of cancer compared with those without cancer after adjustment for baseline CV risk and other potential confounders. We hypothesized that CV risk is increased in all cancer types and is not limited to incident heart failure.

## Methods

We reported this retrospective population-based cohort study according to the Strengthening the Reporting of Observational Studies in Epidemiology guidelines.^6^ The Health Research Ethics Board of Alberta (HREBA.CC-16-0164) provided institutional approval and waived the requirement for participants to provide consent.

### Data sources and cohort

We used an existing database, which incorporates patient registry, physician claims, hospitalizations, and ambulatory care utilization data from all adults registered with Alberta Health (the provincial health ministry) and links it with data from provincial clinical laboratories and vital statistics. This database has been widely used^7^, ^8^, ^9^ because of its population-based coverage of a geographically defined area, including demographic characteristics, health services utilization, and clinical outcomes. Additional information on the database is available elsewhere, including the validation of selected data elements.^10^ All Alberta residents are eligible for insurance coverage by Alberta Health, with >99% participation. The database was used to assemble cohorts of adults who resided in Alberta between April 1, 2007, and December 31, 2018. The index date was April 1, 2007, the day of first contact with Alberta Health, or the participant’s 18th birthday, whichever was latest.

### Cancer, comorbidities, and other characteristics

We linked the primary database with data from the Alberta Cancer Registry to identify participants with cancer as well as cancer stage (0, I, II, III, IV, or not determined), site (breast, melanoma, genitourinary, gynecological, head and neck, hematologic, gastrointestinal, nervous system, thoracic [primarily lung], and other sites), and date of diagnosis. If multiple stages and/or sites were identified within the same participant and incident cancer date, then the highest stage and the most frequent site were recorded. We excluded participants from the cohort if they were diagnosed with cancer between April 1, 2004, and March 31, 2007 (ie, prior to the study period).

We defined other comorbidities using a previously published framework of validated algorithms as applied to Canadian physician claims, hospitalizations, and ambulatory care data, each of which had positive predictive value ≥70% compared with a gold-standard measure such as chart review.^11^ Comorbidities included CV conditions such as atrial fibrillation, heart failure, diabetes, dyslipidemia, hypertension, myocardial infarction (MI), peripheral artery disease, severe obesity, and stroke or transient ischemic attack as well as non-CV conditions such as alcohol misuse, asthma, chronic pain, chronic obstructive pulmonary disease, chronic hepatitis B, cirrhosis, severe constipation, dementia, depression, epilepsy, gout, hypothyroidism, inflammatory bowel disease, irritable bowel syndrome, multiple sclerosis, Parkinson’s disease, peptic ulcer disease, psoriasis, osteoporosis, rheumatoid arthritis, and schizophrenia. Detailed methods for classifying comorbidity status and the specific algorithms used have been previously detailed.^11^ We defined dyslipidemia as an outpatient low-density lipoprotein cholesterol level ≥3.5 mmol/L (135 mg/dL).^12^^,^^13^ Severe obesity was defined using a fee modifier, as in our previous work.^14^ Severe chronic kidney disease was defined by sustained estimated glomerular filtration rate <30 mL/min/1.73 m^2^ and/or registration with a renal replacement program. Participants without data on low-density lipoprotein cholesterol and estimated glomerular filtration rate were considered not to have dyslipidemia and chronic kidney disease, respectively. Each participant was classified with respect to the presence or absence of these 31 chronic conditions at baseline (lookback extended as far as April 1994 when records were available).^15^

As in our prior work, we used administrative data to identify age, biological sex, and rural residence location.^16^ We included the Pampalon index of material deprivation created by Alberta Health Services.^17^^,^^18^ It categorizes participants at the postal code level into 5 bins of socioeconomic inequalities in health care services and population health, with 5 representing the most deprived neighborhoods.

### Outcomes

We assessed all-cause mortality, CV mortality, first MI during follow-up,^11^^,^^19^ first stroke or transient ischemic attack,^11^^,^^20^ new heart failure,^11^^,^^21^ and first pulmonary embolism.^22^ We defined mortality due to CV causes as in previous work^8^ and included International Classification of Diseases-10th Revision codes for ischemic heart disease, stroke, heart failure, valvular heart disease, and arrhythmia. Latest potential follow-up for participants was December 31, 2018.

### Statistical analyses

All analyses were performed using Stata MP version 15.1 (StataCorp). We used time-to-event Weibull survival models to determine the HRs of all outcomes by cancer status (yes or no), time since cancer diagnosis (from diagnosis year to ≥10 years postdiagnosis), cancer stage, and cancer site. We treated cancer as a time-varying covariate; thus, events occurring any time within the study period prior to a cancer diagnosis were included in the no-cancer group and after the cancer diagnosis in the cancer group. We segregated time from cancer diagnosis by the number of years since diagnosis, culminating in 10 or more years from diagnosis, and modeled baseline hazards using restricted cubic splines.^23^

We adjusted for age, sex, neighborhood material deprivation quintile, rural residence, distance to closest cancer center, distance to closest family doctor, and the 31 comorbidities. We determined that the proportional hazard assumption was satisfied by examining plots of the log-negative-log of within-group survivorship probabilities versus log-time. We reported baseline descriptive statistics as counts and percentages or medians with IQRs. We report the number of events and unadjusted and age- and sex-adjusted rates, along with fully adjusted HRs and 95% CIs. The rates per person-year at risk were calculated using Poisson regression.

Missing values occurred in the following variables: material deprivation quintile (14.3%), rural dwelling (12.8%), distance to cancer center (14.2%), family doctor (13.2%), severe obesity (32.9%), and dyslipidemia (36.4%). For the purposes of modeling, we used indicator variables to represent missingness.

### Sensitivity analyses

We performed multiple sensitivity analyses to evaluate the strength of association between cancer and CV events. First, we had planned to model the data using Cox regression, as the computation time for competing risks with the Fine and Gray model was too extensive (more than 2 weeks). Thus, we include the results of Cox modeling as a sensitivity analysis. Second, because death is a competing risk for CV disease, we used a parametric Weibull hazards model with death considered as a competing risk (subdistribution hazards) to determine if the relationship found by censoring at death in our primary analyses provided different results.^23^ Third, we used a subcohort of 1:1 age-sex matched and stratified age analyses to address the imbalance in age between those with cancer and those without cancer (median 56 vs 34 years). A participant who developed cancer was matched within 1 year (of age) to a participant who did not develop cancer. Fourth, we excluded participants diagnosed with cancer at stage 0 (in situ) given that 73% of cases were gynecological and it does not apply to hematologic malignancies. Fifth, we reported associations between cancer site and CV events by biological sex. Sixth, we examined the material deprivation quintile as a possible modifier of the association between cancer and CV outcomes using an interaction term.

## Results

### Characteristics of participants

We identified 4,519,243 participants during the study period (after excluding 34,954 participants because of a history of cancer in the 3 years prior). Of these, 224,016 received new cancer diagnoses during follow-up. Table 1 shows the characteristics of the study populations at first contact. For participants diagnosed with cancer, the median age was 56 years (IQR: 43-67 range) and 57% were women, compared with a median age of 34 years (IQR: 23-49 years) and 49% women among those without cancer. CV disease was prevalent in the cancer group, including 32% with hypertension, 10% with diabetes, 2% with prior MI, and 3% with heart failure. Non-CV disease was also prevalent in the cancer group, including 10% with chronic obstructive pulmonary disease and 8% with depression. Participants without cancer were more than 20 years younger on average, were more likely to be male, and more commonly lived in an urban residence than those without; they also had a lower prevalence of every chronic condition.

**Table 1:** Baseline Characteristics by Cancer Status During Follow-Up

	Cancer (n = 224,016)	No Cancer (n = 4,295,227)
Age, y	56 (43-67)	34 (23-49)
Female	56.8	48.5
Material deprivation quintile	3 (2-4)	3 (2-4)
Rural dwelling	12.1	10.1
Distance to health care, km		
Cancer center	25 (10-55)	25 (10-45)
Family doctor	5 (5-5)	5 (5-5)
Cardiovascular comorbidities		
Dyslipidemia	24.1	22.2
Hypertension	31.7	10.7
Severe obesity	17.1	11.2
Diabetes	10.1	3.6
Prior stroke or transient ischemic attack	4.6	1.6
Heart failure	3.2	1.1
Atrial fibrillation	3.0	1.0
Myocardial infarction	2.1	0.6
Peripheral artery disease	1.0	0.3
Noncardiovascular comorbidities		
Chronic pain	15.1	7.9
Depression	7.8	4.9
COPD	10.1	3.5
Hypothyroidism	7.3	3.2
Osteoporosis	6.9	2.8
Gout	5.8	2.1
Alcohol misuse	2.3	1.2
Asthma	2.1	1.1
Irritable bowel syndrome	1.5	0.8
Epilepsy	1.2	0.7
Rheumatoid arthritis	1.8	0.6
Dementia	0.7	0.6
Schizophrenia	0.7	0.5
Inflammatory bowel disease	0.8	0.4
Multiple sclerosis	0.7	0.3
Severe constipation	0.5	0.3
Psoriasis	0.6	0.3
Severe chronic kidney disease	0.4	0.2
Parkinson’s disease	0.4	0.2
Peptic ulcer disease	0.2	0.1
Cirrhosis	0.2	0.0
Chronic hepatitis B	0.1	0.0

Values are median (IQR) or %. All characteristics were obtained at baseline. Participants in the cancer group developed cancer at some point during follow-up. The following variables were missing data: material deprivation quintile (14.3%), rural dwelling (12.8%), distance to cancer center (14.2%), family doctor (13.2%), severe obesity (32.9%), and dyslipidemia (36.4%).

COPD = chronic obstructive pulmonary disease.

The most frequent cancer sites were gynecological (20%), genitourinary (19%), gastrointestinal (17%), breast (13%), thoracic (10%) and hematologic (9%) (Table 2).

**Table 2:** Site and Stage of Cancers

	Stage						
	All	0	I	II	III	IV	ND
All	224,016	45,230 (20.2)	37,303 (16.7)	37,128 (16.6)	22,795 (10.2)	31,494 (14.1)	50,066 (22.4)
Gynecological	45,534 (20.3)	32,832 (72.1)	4,839 (10.6)	851 (1.9)	1,716 (3.8)	890 (2.0)	4,406 (9.7)
Genitourinary	43,296 (19.3)	3,560 (8.2)	5,997 (13.9)	17,483 (40.4)	3,841 (8.9)	4,237 (9.8)	8,178 (18.9)
Gastrointestinal	36,897 (16.5)	2,314 (6.3)	5,281 (14.3)	6,258 (17.0)	6,715 (18.2)	9,222 (25.0)	7,107 (19.3)
Breast	29,407 (13.1)	3,130 (10.6)	10,163 (34.6)	8,602 (29.3)	2,776 (9.4)	1,268 (4.3)	3,468 (11.8)
Thoracic	21,534 (9.6)	15 (0.1)	3,629 (16.9)	772 (3.6)	4,510 (20.9)	9,555 (44.4)	3,053 (14.2)
Hematological	19,558 (8.7)	0 (0.0)	1,617 (8.3)	1,447 (7.4)	1,312 (6.7)	3,094 (15.8)	12,088 (61.8)
Leukemia	5,632 (2.5)	0 (0.0)	28 (0.5)	39 (0.7)	62 (1.1)	37 (0.7)	5,466 (97.1)
Lymphoma	8,113 (3.6)	0 (0.0)	1,580 (19.5)	1,406 (17.3)	1,248 (15.4)	3,051 (37.6)	828 (10.2)
Other	5,813 (2.6)	0 (0.0)	9 (0.2)	2 (0.0)	2 (0.0)	6 (0.1)	5,794 (99.7)
Melanoma	10,140 (4.5)	3,203 (31.6)	1,907 (18.8)	721 (7.1)	590 (5.8)	234 (2.3)	3,485 (34.4)
Head and neck	4,589 (2.1)	175 (3.8)	691 (15.1)	387 (8.4)	488 (10.6)	2,133 (46.5)	715 (15.6)
Nervous system	2,553 (1.1)	0 (0.0)	6 (0.2)	9 (0.4)	6 (0.2)	6 (0.2)	2,526 (98.9)
Other	10,508 (4.7)	1 (0.0)	3,173 (30.2)	598 (5.7)	841 (8.0)	855 (8.1)	5,040 (48.0)

Values are n (%). The all-stages column shows the percentage of each cancer site. The stage-specific columns show the percentage of each stage within each cancer site.

ND = not determined

### Outcomes

Median follow-up duration was 11.8 years (IQR: 6.4-11.8 years). During follow-up, there were 248,541 deaths, including 73,360 from CV causes. Nonfatal CV outcomes included 123,342 with incident heart failure, 53,496 with acute MI, 178,433 with stroke, and 115,210 with pulmonary embolism. In the cancer groups, the highest rates of CV events were as follows: hematologic cancers for CV deaths (3.7 per 1,000 participant-years), genitourinary cancers for acute MIs (2.4 per 1,000 participant-years), nervous system cancers in stroke (16.4 per 1,000 participant-years), hematologic cancers in heart failure (12.0 per 1,000 participant-years), and nervous system cancers in pulmonary embolism (16.5 per 1,000 participant-years). High rates of CV events were also noted for thoracic cancers (Supplemental Table 1).

After adjustment for age and sex, the rate of CV death in the cancer group was 3.0 per 1,000 patient-years compared with 2.9, 3.8, and 6.8 per 1,000 patient-years for participants with hypertension, diabetes, or prior MI, respectively (Supplemental Table 2).

After adjustment for baseline covariates, participants with cancer had HRs of 1.33 (95% CI: 1.29-1.37) for CV mortality, 1.01 (95% CI: 0.97-1.05) for acute MI, 1.44 (95% CI: 1.41-1.47) for stroke, 1.62 (95% CI: 1.59-1.65) for heart failure, and 3.43 (95% CI: 3.37-3.50) for pulmonary embolism, all compared with participants without cancer (Table 3). When we subcategorized stroke as hemorrhagic stroke and ischemic stroke, the HRs among participants with cancer (vs those without) were 1.41 (95% CI: 1.38-1.44) for hemorrhagic stroke and 1.51 (95% CI: 1.48-1.54) for ischemic stroke.

**Table 3:** Adjusted HRs for Cardiovascular Outcomes by Cancer Status: Primary and Sensitivity Analyses

	All-Cause Mortality	CV Mortality	Acute MI	Stroke	Heart Failure	Pulmonary Embolism
Weibull RCS (primary)						
Cancer (reference no cancer)	8.34 (8.26-8.42)	1.33 (1.29-1.37)	1.01 (0.97-1.05)	1.44 (1.41-1.47)	1.62 (1.59-1.65)	3.43 (3.37-3.50)
Competing risks (sensitivity)						
Cancer (reference no cancer)			1.05 (1.01-1.09)	1.40 (1.38-1.43)	1.79 (1.75-1.82)	2.77 (2.72-2.82)
Cox (sensitivity)						
Cancer (reference no cancer)	8.34 (8.26-8.42)	1.33 (1.29-1.37)	1.01 (0.97-1.04)	1.44 (1.41-1.47)	1.62 (1.59-1.65)	3.43 (3.37-3.50)
Age and sex 1:1 matched (sensitivity)						
Cancer (reference no cancer)	12.92 (12.72-13.13)	1.73 (1.67-1.80)	1.03 (0.99-1.08)	1.38 (1.35-1.41)	1.59 (1.55-1.62)	3.13 (3.05-3.20)
Exclude participants diagnosed with stage 0 cancer (sensitivity)						
Cancer (reference no cancer)	9.22 (9.13-9.31)	1.36 (1.32-1.40)	1.03 (0.99-1.07)	1.49 (1.46-1.52)	1.69 (1.66-1.73)	3.97 (3.89-4.04)

Values are HR (95% CI). Reference group is the no-cancer group in all rows (HR: 1.00). Fully adjusted for baseline age, biological sex, neighborhood material deprivation quintile, rural or urban, distance to cancer center, and distance to family doctor, plus 31 comorbidities: alcohol misuse, asthma, atrial fibrillation, heart failure, severe chronic kidney disease, chronic pain, chronic pulmonary disease, dyslipidemia, viral hepatitis B, cirrhosis, severe constipation, dementia, depression, diabetes mellitus, epilepsy, gout, hypertension, hypothyroidism, inflammatory bowel disease, irritable bowel syndrome, myocardial infarction, multiple sclerosis, severe obesity, osteoporosis, Parkinson’s disease, peptic ulcer disease, peripheral artery disease, psoriasis, rheumatoid arthritis, schizophrenia, and stroke or transient ischemic attack.

CV = cardiovascular; MI = myocardial infarction; RCS = restricted cubic splines.

### Sensitivity analyses

Results from the Cox model and the Weibull model were very similar to the primary results (Table 3). The results were also similar when we excluded participants <50 years of age and when we excluded those diagnosed with stage 0 cancers. Analyses that treated mortality as a competing risk found that the risks for acute MI, stroke, and heart failure associated with cancer were similar to those from the primary analysis, although the risk for pulmonary embolism was slightly attenuated (from HR: 3.43 [95% CI: 3.37-3.50] to HR: 2.77 [95% CI: 2.72-2.82]).

Supplemental Table 3 shows that the risk for CV outcomes associated with cancer varied significantly by socioeconomic status. The excess risk for all-cause mortality (*P* < 0.001) and pulmonary embolism (*P* = 0.013) associated with cancer was smaller for participants in the least deprived neighborhoods, although their risk for heart failure was greater (*P* = 0.011).

Risks for CV mortality (HR: 1.02-3.24), pulmonary embolism (HR: 1.64-18.75), heart failure (HR: 1.00-3.11), and stroke (HR: 1.16-11.20) among participants with cancer were greater than among control subjects without cancer for all cancer sites except melanoma (Table 4). Two cancer sites, thoracic and hematologic, were also at greater risk for acute MI (HR: 1.60 [95% CI: 1.41-1.82] and HR: 1.23 [95% CI: 1.11-1.37], respectively). CV risks by cancer sites were qualitatively similar between the sexes.

**Table 4:** Adjusted HRs for Cardiovascular Outcomes by Cancer Site and Biological Sex

	All-Cause Mortality	CV Mortality	Acute MI	Stroke	Heart Failure	Pulmonary Embolism
Cancer (reference no cancer)						
Gynecological	5.92 (5.71-6.13)	1.17 (1.02-1.35)	0.87 (0.73-1.04)	1.22 (1.14-1.30)	1.19 (1.10-1.29)	2.26 (2.14-2.38)
Genitourinary	3.73 (3.65-3.81)	1.20 (1.14-1.27)	0.96 (0.90-1.02)	1.31 (1.27-1.36)	1.29 (1.24-1.33)	2.26 (2.17-2.35)
Gastrointestinal	13.04 (12.84-13.25)	1.39 (1.31-1.48)	1.00 (0.91-1.09)	1.43 (1.37-1.50)	1.73 (1.66-1.81)	5.37 (5.18-5.56)
Breast	3.48 (3.38-3.59)	1.14 (1.05-1.23)	0.98 (0.87-1.10)	1.16 (1.10-1.22)	1.26 (1.20-1.33)	2.65 (2.53-2.77)
Thoracic	31.16 (30.65-31.68)	1.87 (1.71-2.05)	1.60 (1.41-1.82)	2.54 (2.39-2.69)	3.11 (2.94-3.29)	9.20 (8.79-9.64)
Hematological	7.69 (7.51-7.87)	1.59 (1.48-1.71)	1.23 (1.11-1.37)	1.72 (1.63-1.82)	2.89 (2.76-3.02)	4.73 (4.51-4.96)
Leukemia	7.01 (6.70-7.32)	1.45 (1.26-1.67)	1.21 (1.01-1.46)	1.57 (1.43-1.73)	2.49 (2.29-2.71)	4.23 (3.87-4.63)
Lymphoma	7.55 (7.26-7.85)	1.38 (1.21-1.57)	1.05 (0.88-1.26)	1.58 (1.45-1.72)	2.63 (2.44-2.83)	4.79 (4.46-5.15)
Other	8.50 (8.18-8.84)	1.96 (1.74-2.21)	1.50 (1.26-1.79)	2.07 (1.89-2.26)	3.63 (3.38-3.90)	5.17 (4.76-5.63)
Melanoma	2.85 (2.71-3.00)	1.02 (0.90-1.16)	0.75 (0.63-0.89)	1.26 (1.16-1.37)	1.00 (0.91-1.11)	1.64 (1.49-1.82)
Head and neck	9.41 (8.98-9.88)	1.93 (1.64-2.26)	0.91 (0.71-1.16)	1.57 (1.39-1.77)	1.73 (1.53-1.96)	3.55 (3.16-3.98)
Nervous system	76.52 (73.00-80.21)	3.24 (2.22-4.73)	1.20 (0.68-2.11)	11.20 (9.84-12.74)	2.61 (1.96-3.48)	18.75 (16.77-20.97)
Other	15.32 (14.82-15.83)	1.43 (1.21-1.68)	0.71 (0.55-0.91)	1.50 (1.35-1.65)	1.56 (1.40-1.74)	3.62 (3.33-3.93)
Women						
Cancer (reference no cancer)						
Gynecological	6.38 (6.16-6.62)	1.26 (1.09-1.44)	1.00 (0.84-1.19)	1.25 (1.17-1.33)	1.28 (1.18-1.39)	2.24 (2.12-2.36)
Genitourinary	7.01 (6.66-7.38)	1.34 (1.13-1.58)	1.41 (1.11-1.78)	1.56 (1.39-1.74)	1.80 (1.62-2.00)	3.44 (3.11-3.80)
Gastrointestinal	13.31 (13.00-13.62)	1.31 (1.18-1.45)	1.09 (0.92-1.28)	1.49 (1.39-1.60)	1.75 (1.63-1.87)	5.24 (4.97-5.53)
Breast	3.48 (3.37-3.58)	1.13 (1.04-1.22)	0.95 (0.84-1.06)	1.18 (1.12-1.24)	1.26 (1.20-1.33)	2.73 (2.61-2.86)
Thoracic	30.54 (29.82-31.28)	1.85 (1.61-2.12)	2.32 (1.94-2.76)	2.67 (2.47-2.90)	3.28 (3.03-3.55)	8.93 (8.39-9.52)
Hematological	7.32 (7.06-7.60)	1.42 (1.25-1.60)	1.34 (1.11-1.62)	1.75 (1.62-1.90)	2.92 (2.72-3.12)	4.47 (4.15-4.80)
Melanoma	2.57 (2.36-2.79)	0.87 (0.70-1.08)	0.88 (0.64-1.21)	1.21 (1.07-1.37)	1.05 (0.90-1.22)	1.71 (1.48-1.97)
Head and neck	9.50 (8.67-10.40)	1.95 (1.43-2.64)	1.15 (0.65-2.02)	1.52 (1.20-1.92)	1.97 (1.57-2.49)	3.59 (2.89-4.45)
Nervous system	72.89 (67.60-78.59)	3.90 (2.31-6.59)	1.19 (0.39-3.71)	10.23 (8.35-12.54)	2.15 (1.30-3.57)	13.83 (11.41-16.77)
Other	15.70 (14.99-16.44)	1.21 (0.93-1.57)	0.94 (0.66-1.34)	1.52 (1.33-1.73)	1.58 (1.36-1.84)	3.35 (3.00-3.73)
Men						
Cancer (reference no cancer)						
Genitourinary	3.46 (3.38-3.55)	1.22 (1.15-1.28)	0.98 (0.92-1.05)	1.28 (1.24-1.33)	1.25 (1.21-1.30)	2.09 (2.00-2.18)
Gastrointestinal	12.92 (12.66-13.19)	1.45 (1.34-1.58)	0.98 (0.88-1.09)	1.39 (1.31-1.47)	1.72 (1.63-1.82)	5.43 (5.19-5.69)
Breast	4.31 (3.26-5.70)	1.53 (0.80-2.94)	0.75 (0.24-2.32)	1.53 (0.91-2.59)	1.42 (0.79-2.56)	2.23 (1.16-4.29)
Thoracic	32.02 (31.30-32.76)	1.91 (1.68-2.16)	1.23 (1.03-1.48)	2.42 (2.22-2.63)	2.98 (2.74-3.22)	9.63 (9.01-10.30)
Hematological	7.96 (7.72-8.21)	1.72 (1.56-1.89)	1.22 (1.08-1.38)	1.70 (1.58-1.82)	2.87 (2.70-3.04)	4.91 (4.61-5.23)
Melanoma	3.10 (2.90-3.31)	1.16 (0.99-1.35)	0.72 (0.59-0.90)	1.31 (1.17-1.45)	1.00 (0.88-1.13)	1.58 (1.38-1.82)
Head and neck	9.26 (8.75-9.79)	1.90 (1.58-2.30)	0.87 (0.67-1.14)	1.56 (1.36-1.80)	1.64 (1.41-1.90)	3.41 (2.98-3.91)
Nervous system	77.44 (72.90-82.26)	2.71 (1.58-4.68)	1.14 (0.59-2.18)	11.79 (9.98-13.93)	2.81 (1.99-3.98)	22.66 (19.75-26.01)
Other	15.38 (14.68-16.12)	1.70 (1.38-2.09)	0.60 (0.42-0.84)	1.50 (1.29-1.74)	1.60 (1.37-1.87)	4.17 (3.67-4.73)

Values are HR (95% CI). Reference group is the no-cancer group in all rows (HR: 1.0). Adjusted for baseline age, biological sex, neighborhood material deprivation quintile, rural or urban, distance to cancer center, and distance to family doctor, plus 31 comorbidities: alcohol misuse, asthma, atrial fibrillation, heart failure, severe chronic kidney disease, chronic pain, chronic pulmonary disease, dyslipidemia, viral hepatitis B, cirrhosis, severe constipation, dementia, depression, diabetes mellitus, epilepsy, gout, hypertension, hypothyroidism, inflammatory bowel disease, irritable bowel syndrome, myocardial infarction, multiple sclerosis, severe obesity, osteoporosis, Parkinson’s disease, peptic ulcer disease, peripheral artery disease, psoriasis, rheumatoid arthritis, schizophrenia, and stroke or transient ischemic attack.

AMI = acute myocardial infarction; CV = cardiovascular.

The excess CV risk associated with cancer was greatest during the first year following the cancer diagnosis for all outcomes (HR: 1.24-8.36) and declined over time, althoughit remained significantly elevated for CV mortality, heart failure, and pulmonary embolism even after 10 years of follow-up (Central Illustration, Supplemental Table 4). Similarly, participants with more advanced cancer were at higher risk for CV outcomes (Supplemental Table 5). However, even patients with very early stage disease (stages 0 and I) had higher risk for CV events relative to control subjects without cancer.

**Central Illustration:**
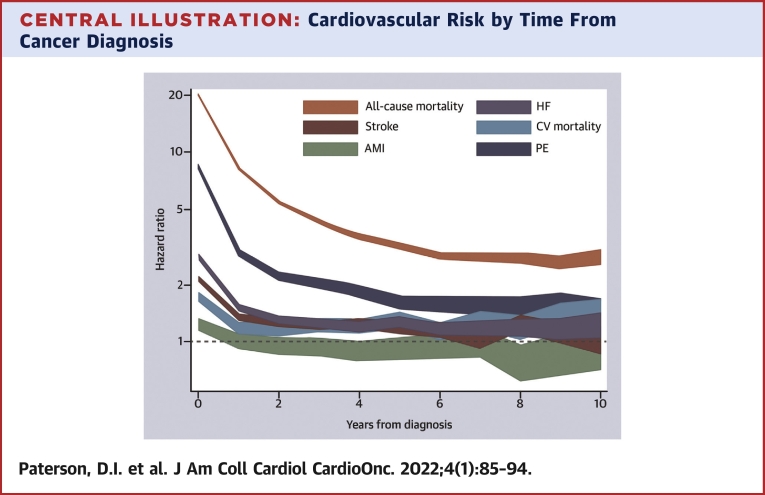
Cardiovascular Risk by Time From Cancer Diagnosis In this population-based cohort study of 4,519,243 adults, we determined that a new cancer diagnosis was independently associated with a significantly higher early and persistent risk for cardiovascular death, pulmonary embolism, heart failure, stroke, and acute myocardial infarction. The risk for cardiovascular death, pulmonary embolism, and heart failure was attenuated but remained significantly elevated after 10 years of follow-up. The width of the lines shows the 95% CI. Specific point and interval estimates are available in Supplemental Table 4.

Supplemental Table 6 shows the baseline characteristics of participants with and without cancer after matching for age and sex. After matching, the excess risks for nonfatal CV events associated with cancer were similar to those in the primary analysis (Table 3), and the excess risks for all-cause and CV mortality associated with cancer were accentuated (from HR: 8.34 [95% CI: 8.26-8.42] to HR: 12.92 [95% CI: 12.72-13.13]; and from HR: 1.33 [95% CI: 1.29-1.37] to HR: 1.73 [95% CI: 1.67-1.80]). Supplemental Table 7 demonstrates that the risk for CV events in patients with cancer varies by age at cancer diagnosis. For all outcomes except MI, the excess risk was higher among younger participants.

## Discussion

In this large population-based study, we found that a new diagnosis of cancer was associated with an increased risk for fatal and nonfatal CV events, even after adjustment for baseline risk. Regardless of cancer site, patients were at increased risk for CV mortality, heart failure, stroke, or pulmonary embolism, and this risk persisted to 10 years for heart failure and pulmonary embolism (Central Illustration). Genitourinary, thoracic, hematologic, gastrointestinal, and nervous system cancers were identified as higher risk groups warranting further study.

### Previous population-based studies of CV outcomes in patients with cancer

Our study has several novel aspects compared with prior work. First, ours is a uniquely comprehensive study of CV risk in cancer. We have included data on cancer site, stage, and time from diagnosis in addition to evaluating several CV outcomes of interest. Prior studies of incident CV disease in cancer have often included only 1 cancer site, usually breast cancer, and have studied only CV mortality and/or incident heart failure.^3^^,^^4^^,^^24^ Second, population-based studies have shown that cancer is associated with higher risk for venous and arterial thromboembolism during the first year from cancer diagnosis,^25^ whereas our findings demonstrate that the excess risk extends to year 7 for stroke and past year 10 for heart failure and pulmonary embolism. Furthermore, prior survival analyses have adjusted only for age and sex but not other risk factors for incident CV disease. Our study demonstrates that patients with cancer were at higher risk for 5 distinct CV events, while controlling for sociodemographic data and 31 clinical covariates. Similar to Sturgeon et al,^3^ we found that the first year from cancer diagnosis was associated with the greatest excess risk for CV mortality. However, we also found an excess of nonfatal CV events during this period, which suggests that patients with cancer may benefit from comanagement that includes cardiologists as well as stroke and thrombosis specialists.^26^

### Is cancer a risk factor for CV disease?

Despite adjustment for covariates including age, sex, hypertension, diabetes, dyslipidemia, and prior CV disease, a new cancer diagnosis was associated with a higher risk for CV mortality, acute MI, stroke, heart failure, and pulmonary embolism. Although data were not available on smoking status, this characteristic is unlikely to account for the observed HR of 1.22 for CV mortality and 1.55 for stroke, given that the median prevalence of smoking in Canada was 20% among patients with cancer versus 19% among those without cancer during the study period.^27^ Another notable finding was that the HR for incident heart failure was 1.62, after adjustment for all major risk factors of heart failure (age, hypertension, diabetes, and prior MI).

We recently reported that patients with cancer have relative cardiac hypertrophy prior to receiving cancer treatment,^28^ and others have reported abnormal cardiac function at baseline.^29^ Thus, patients with cancer may be predisposed to developing CV disease independent of treatment. Indeed, a recent study using data from the SEER database found that patients with breast cancer not exposed to chemotherapy or radiotherapy were at higher risk for incident CV disease compared with the general population, especially without tumor resection.^30^

### High-risk cancer sites

Most studies of adverse CV outcomes in cancer have reported on participants with breast cancer. Our findings suggest that the risk for incident CV disease is higher for other cancer sites, notably genitourinary, thoracic, hematologic, gastrointestinal, and nervous system. Participants with these cancers constituted 55% of the cancer cohort and accounted for more than 71% of the incident CV burden. Furthermore, the majority of these people with cancer will experience extended survival, with 5-year survival rates of 93% for prostate cancer and 44% to 86% for hematologic malignancies.^31^ Although median survival for lung cancer remains significantly lower than for these other forms of cancer, immune-modifying therapies now provide long-term survival in a substantial subset of patients. Thus, future work should further elucidate the CV risk in patients with these understudied cancer types.

### Study limitations

Although the cohort that we used included detailed information on sociodemographic factors and a detailed assessment of comorbidity, data on cancer therapies, patient ethnicity and some risk factors for atherosclerosis such as smoking and physical activity were not available. However, we found that the adjusted CV risk was elevated for all cancer stages, including patients with very early stage disease who were less likely to receive radiation and/or systemic treatment. Regardless, our study shows that patients with prior cancer are susceptible to a variety of CV events over a long time frame. Unfortunately, this risk is unlikely to diminish in the short term given that many newer cancer therapies are also associated with increased risk for myocardial injury and heart failure. Future work should evaluate CV events in large prospective cancer registries with enhanced phenotyping and risk modeling. Such work would potentially lead to better prediction of CV risk for patients with cancer and survivors and improved prevention and treatment strategies.

## Conclusions

In this population-based study of 4,519,243 participants with median follow-up of nearly 12 years, we found that a new cancer diagnosis was independently associated with a significantly higher risk for CV death, stroke, heart failure, and pulmonary embolism, especially in the first year. This risk was relatively more pronounced in participants with genitourinary, thoracic, hematologic, and nervous system cancers. Future studies should evaluate other potential contributors to CV risk, including cancer therapies and emerging risk factors of cardiotoxicity.

Perspectives **COMPETENCY IN MEDICAL KNOWLEDGE:** Patients with cancer are at high risk for fatal and nonfatal CV events, including heart failure, stroke, and pulmonary embolism. Patients with genitourinary gastrointestinal, hematologic, nervous system, or thoracic malignancies accounted for the majority of CV events and were higher risk cancer subtypes.

**TRANSLATIONAL OUTLOOK:** Large prospective registries are needed to enhance patient phenotyping and to elucidate the impact of race, ethnicity, and socioeconomic factors as well as cancer treatments on CV outcomes.

## Funding Support and Author Disclosures

Research support was provided (to Dr Tonelli) by a foundation grant from the Canadian Institutes of Health Research. Dr Tonelli was supported by the David Freeze Chair in Health Services Research at the University of Calgary. The sponsors had no role in the design and conduct of the study; collection, management, analysis, and interpretation of the data; preparation, review, or approval of the paper; or decision to submit the manuscript for publication. This study is based in part by data provided by Alberta Health and Alberta Health Services. The interpretation and conclusions contained herein are those of the researchers and do not represent the views of the government of Alberta or Alberta Health Services. Neither the government of Alberta nor Alberta Health or Alberta Health Services expresses any opinion in relation to this study. The authors have reported that they have no relationships relevant to the contents of this paper to disclose.
