# Supplementary material for: Incident Cardiovascular Disease Among Adults With Cancer: A Population-Based Cohort Study
Source: JACC CardioOncol. 2022 Mar 15;4(1):85–94. doi: 10.1016/j.jaccao.2022.01.100 (PMC9040097; doi:10.1016/j.jaccao.2022.01.100)
Supplement: Supplemental Tables 1–7 [file mmc1.docx]

**Supplemental Table 1. Mortality and cardiovascular events by cancer site**

| **Cancer** | **All-cause mortality** | **CV**  **mortality** | **AMI** | **Stroke** | **Heart**  **failure** | **Pulmonary**  **embolism** |
| --- | --- | --- | --- | --- | --- | --- |
|  | **Events (rate)** | **Events (rate)** | **Events (rate)** | **Events (rate)** | **Events (rate)** | **Events (rate)** |
| No cancer | 179,205 (4.6) | 68,312 (1.7) | 50,648 (1.3) | 166,841 (4.2) | 111,980 (2.8) | 100,649 (2.5) |
| Cancer | 69,336 (31.6) | 5,048 (2.3) | 2,848 (1.3) | 11,592 (5.7) | 11,362 (5.6) | 14,561 (7.0) |
| Gynecological | 3,180 (6.6) | 199 (0.4) | 126 (0.3) | 902 (1.9) | 581 (1.2) | 1,474 (3.1) |
| Genitourinary | 9,809 (21.6) | 1,504 (3.3) | 1,079 (2.4) | 3,215 (7.7) | 2,993 (7.2) | 2,584 (6.0) |
| Gastrointestinal | 19,366 (59.5) | 979 (3.0) | 492 (1.6) | 1,906 (6.4) | 2,091 (7.2) | 3,313 (11.0) |
| Breast | 4,546 (14.3) | 647 (2.0) | 285 (0.9) | 1,541 (5.2) | 1,403 (4.7) | 1,946 (6.4) |
| Thoracic | 16,637 (104.7) | 460 (2.9) | 242 (1.6) | 1,142 (8.2) | 1,247 (9.2) | 1,903 (13.1) |
| Hematological | 7,201 (38.3) | 695 (3.7) | 362 (2.0) | 1,408 (8.3) | 2,008 (12.0) | 1,787 (10.3) |
| Melanoma | 1,432 (13.0) | 243 (2.2) | 123 (1.1) | 591 (5.7) | 414 (4.0) | 386 (3.6) |
| Head and Neck | 1,716 (39.0) | 150 (3.4) | 65 (1.5) | 265 (6.5) | 250 (6.1) | 296 (7.0) |
| Nervous System | 1,760 (89.2) | 27 (1.4) | 12 (0.6) | 231 (16.4) | 47 (2.5) | 308 (16.5) |
| Other | 3,689 (37.9) | 144 (1.5) | 62 (0.6) | 391 (4.3) | 328 (3.6) | 564 (6.1) |
|  |  |  |  |  |  |  |

AMI acute myocardial infarction, CI confidence interval, CV cardiovascular, pys participant-years

N (rate per 1000 pys)

**Supplemental Table 2. Unadjusted and age-sex adjusted rates by cancer, diabetes and prior AMI status over time to first event (single CV event)**

| **Cohort** | **Events** | **1000 pys** | **Unadjusted rate**  **(per 1000 pys)** | **Age-sex^1^ adjusted rate**  **(per 1000 pys)** |
| --- | --- | --- | --- | --- |
| *All-cause mortality* | | | | |
| Cancer | 69,336 | 920 | 75.3 (74.8,75.9) | 39.6 (39.3,39.9) |
| Hypertension | 180,045 | 8,084 | 22.3 (22.2,22.4) | 8.6 (8.6,8.7) |
| Diabetes | 77,374 | 3,044 | 25.4 (25.2,25.6) | 11.1 (11.0,11.2) |
| Prior AMI | 25,514 | 487 | 52.4 (51.7,53.0) | 14.4 (14.2,14.6) |
| *CV mortality* | | | | |
| Cancer | 5,048 | 920 | 5.5 (5.3,5.6) | 3.0 (2.9,3.1) |
| Hypertension | 62,098 | 8,084 | 7.7 (7.6,7.7) | 2.9 (2.8,2.9) |
| Diabetes | 25,993 | 3,044 | 8.5 (8.4,8.6) | 3.8 (3.7,3.8) |
| Prior AMI | 12,729 | 487 | 26.1 (25.7,26.6) | 6.8 (6.7,7.0) |
| *AMI* | | | | |
| Cancer | 2,848 | 905 | 3.1 (3.0,3.3) | 1.7 (1.6,1.8) |
| Hypertension | 34,186 | 7,880 | 4.3 (4.3,4.4) | 2.0 (2.0,2.0) |
| Diabetes | 15,427 | 2,961 | 5.2 (5.1,5.3) | 2.4 (2.4,2.4) |
| Prior AMI | 4,079 | 247 | 16.5 (16.0,17.0) | 4.2 (4.1,4.3) |
| *Stroke* | | | | |
| Cancer | 11,592 | 861 | 13.5 (13.2,13.7) | 7.8 (7.6,7.9) |
| Hypertension | 111,607 | 7,533 | 14.8 (14.7,14.9) | 6.8 (6.8,6.9) |
| Diabetes | 42,981 | 2,833 | 15.2 (15.0,15.3) | 7.4 (7.3,7.5) |
| Prior AMI | 11,400 | 431 | 26.4 (25.9,26.9) | 9.0 (8.8,9.1) |
| *Heart failure* | | | | |
| Cancer | 11,362 | 862 | 13.2 (12.9,13.4) | 7.0 (6.9,7.2) |
| Hypertension | 93,720 | 7,391 | 12.7 (12.6,12.8) | 5.2 (5.2,5.2) |
| Diabetes | 39,554 | 2,739 | 14.4 (14.3,14.6) | 6.6 (6.5,6.6) |
| Prior AMI | 13,061 | 346 | 37.7 (37.1,38.4) | 11.9 (11.7,12.1) |
| *Pulmonary embolism* | | | | |
| Cancer | 14,561 | 868 | 16.8 (16.5,17.0) | 10.7 (10.5,10.9) |
| Hypertension | 56,461 | 7,823 | 7.2 (7.2,7.3) | 4.0 (3.9,4.0) |
| Diabetes | 22,273 | 2,939 | 7.6 (7.5,7.7) | 4.3 (4.2,4.3) |
| Prior AMI | 5,090 | 464 | 11.0 (10.7,11.3) | 4.9 (4.8,5.1) |

^1^Adjusted for age and sex in the whole study population

AMI acute myocardial infarction, CV cardiovascular, pys participant-years

The rates are presented with 95% confidence intervals. The cohort groups are not mutually exclusive.

**Supplemental Table 3. Cardiovascular risk by cancer status within material deprivation quintiles**

| **Cancer stage** | **All-cause mortality** | **CV mortality** | **AMI** | **Stroke** | **Heart**  **failure** | **Pulmonary**  **embolism** |
| --- | --- | --- | --- | --- | --- | --- |
|  | **HR (95% CI)** | **HR (95% CI)** | **HR (95% CI)** | **HR (95% CI)** | **HR (95% CI)** | **HR (95% CI)** |
| Cancer vs no cancer |  |  |  |  |  |  |
| 1 – least deprived | 7.60 (7.43,7.77) | 1.34 (1.25,1.43) | 0.94 (0.85,1.04) | 1.46 (1.40,1.53) | 1.70 (1.62,1.78) | 3.19 (3.06,3.33) |
| 2 | 8.15 (7.96,8.34) | 1.28 (1.19,1.38) | 1.02 (0.92,1.12) | 1.48 (1.41,1.55) | 1.66 (1.58,1.74) | 3.36 (3.22,3.51) |
| 3 | 8.15 (7.98,8.32) | 1.28 (1.19,1.37) | 0.93 (0.85,1.02) | 1.40 (1.34,1.47) | 1.61 (1.54,1.68) | 3.41 (3.28,3.55) |
| 4 | 8.35 (8.19,8.51) | 1.33 (1.25,1.42) | 1.06 (0.98,1.14) | 1.42 (1.36,1.47) | 1.55 (1.49,1.62) | 3.45 (3.32,3.58) |
| 5 – most deprived | 8.27 (8.12,8.43) | 1.33 (1.25,1.41) | 1.03 (0.95,1.11) | 1.38 (1.33,1.44) | 1.54 (1.48,1.61) | 3.52 (3.39,3.65) |
| P for difference | <0.001 | 0.775 | 0.170 | 0.179 | 0.011 | 0.013 |
|  |  |  |  |  |  |  |

AMI acute myocardial infarction, CI confidence interval, CV cardiovascular, HR hazards ratio, TIA transient ischemic attack

Adjusted for baseline age, biological sex, rural/urban, distances to cancer centre, and distance to family doctor, plus 31 comorbidities: alcohol misuse, asthma, atrial fibrillation, heart failure, severe chronic kidney disease, chronic pain, chronic pulmonary disease, dyslipidemia, viral hepatitis B, cirrhosis, severe constipation, dementia, depression, diabetes mellitus, epilepsy, gout, hypertension, hypothyroidism, inflammatory bowel disease, irritable bowel syndrome, myocardial infarction, multiple sclerosis, severe obesity, osteoporosis, Parkinson’s disease, peptic ulcer disease, peripheral artery disease, psoriasis, rheumatoid arthritis, schizophrenia, and stroke/TIA.

**Supplemental Table 4. Cardiovascular risk by time from cancer diagnosis**

| **Cancer** | **All-cause mortality** | **CV mortality** | **AMI** | **Stroke** | **Heart**  **failure** | **Pulmonary**  **embolism** |
| --- | --- | --- | --- | --- | --- | --- |
|  | **HR (95% CI)** | **HR (95% CI)** | **HR (95% CI)** | **HR (95% CI)** | **HR (95% CI)** | **HR (95% CI)** |
| No cancer | 1.00 | 1.00 | 1.00 | 1.00 | 1.00 | 1.00 |
| Cancer, time from diagnosis |  |  |  |  |  |  |
| Year 0-1 | 20.03 (19.80,20.27) | 1.73 (1.64,1.83) | 1.24 (1.15,1.33) | 2.14 (2.08,2.22) | 2.81 (2.72,2.90) | 8.36 (8.16,8.56) |
| Year 1-2 | 8.22 (8.06,8.38) | 1.19 (1.10,1.28) | 1.00 (0.92,1.10) | 1.35 (1.29,1.42) | 1.52 (1.45,1.59) | 2.93 (2.81,3.06) |
| Year 2-3 | 5.45 (5.31,5.59) | 1.16 (1.07,1.26) | 0.95 (0.86,1.06) | 1.26 (1.20,1.33) | 1.31 (1.24,1.38) | 2.22 (2.10,2.35) |
| Year 3-4 | 4.31 (4.18,4.44) | 1.23 (1.13,1.34) | 0.94 (0.84,1.05) | 1.23 (1.16,1.30) | 1.26 (1.18,1.33) | 2.04 (1.91,2.17) |
| Year 4-5 | 3.60 (3.47,3.74) | 1.22 (1.11,1.33) | 0.89 (0.79,1.01) | 1.26 (1.18,1.34) | 1.21 (1.13,1.29) | 1.85 (1.73,1.99) |
| Year 5-6 | 3.23 (3.10,3.37) | 1.30 (1.18,1.44) | 0.92 (0.80,1.06) | 1.18 (1.10,1.27) | 1.28 (1.19,1.37) | 1.62 (1.49,1.76) |
| Year 6-7 | 2.85 (2.71,2.99) | 1.14 (1.02,1.28) | 0.94 (0.81,1.09) | 1.14 (1.05,1.24) | 1.17 (1.08,1.27) | 1.59 (1.45,1.75) |
| Year 7-8 | 2.77 (2.62,2.93) | 1.29 (1.14,1.46) | 0.98 (0.83,1.16) | 1.02 (0.92,1.13) | 1.19 (1.08,1.30) | 1.55 (1.39,1.72) |
| Year 8-9 | 2.76 (2.59,2.94) | 1.19 (1.03,1.38) | 0.78 (0.62,0.97) | 1.25 (1.13,1.39) | 1.20 (1.07,1.33) | 1.53 (1.35,1.73) |
| Year 9-10 | 2.65 (2.44,2.87) | 1.36 (1.15,1.61) | 0.86 (0.66,1.11) | 1.11 (0.97,1.28) | 1.16 (1.02,1.33) | 1.56 (1.34,1.81) |
| ≥10 years post | 2.80 (2.54,3.08) | 1.37 (1.11,1.69) | 0.96 (0.71,1.31) | 1.02 (0.86,1.23) | 1.23 (1.04,1.44) | 1.37 (1.11,1.68) |
|  |  |  |  |  |  |  |

AMI acute myocardial infarction, CI confidence interval, CV cardiovascular, HR hazards ratio, TIA transient ischemic attack

Adjusted for baseline age, biological sex, neighbourhood material deprivation quintile, rural/urban, distances to cancer centre, and distance to family doctor, plus 31 comorbidities: alcohol misuse, asthma, atrial fibrillation, heart failure, severe chronic kidney disease, chronic pain, chronic pulmonary disease, dyslipidemia, viral hepatitis B, cirrhosis, severe constipation, dementia, depression, diabetes mellitus, epilepsy, gout, hypertension, hypothyroidism, inflammatory bowel disease, irritable bowel syndrome, myocardial infarction, multiple sclerosis, severe obesity, osteoporosis, Parkinson’s disease, peptic ulcer disease, peripheral artery disease, psoriasis, rheumatoid arthritis, schizophrenia, and stroke/TIA.

**Supplemental Table 5. Cardiovascular risk by initial cancer stage status**

| **Cancer stage** | **All-cause mortality** | **CV mortality** | **AMI** | **Stroke** | **Heart**  **failure** | **Pulmonary**  **embolism** |
| --- | --- | --- | --- | --- | --- | --- |
|  | **HR (95% CI)** | **HR (95% CI)** | **HR (95% CI)** | **HR (95% CI)** | **HR (95% CI)** | **HR (95% CI)** |
| No cancer | 1.00 | 1.00 | 1.00 | 1.00 | 1.00 | 1.00 |
| 0 | 1.78 (1.70,1.87) | 1.01 (0.92,1.11) | 0.80 (0.71,0.91) | 1.15 (1.08,1.21) | 1.07 (1.00,1.14) | 1.29 (1.22,1.37) |
| I | 3.45 (3.37,3.54) | 1.17 (1.10,1.25) | 1.00 (0.92,1.09) | 1.24 (1.19,1.29) | 1.26 (1.21,1.32) | 2.17 (2.08,2.26) |
| II | 3.58 (3.50,3.66) | 1.20 (1.14,1.27) | 0.92 (0.86,0.98) | 1.26 (1.21,1.30) | 1.30 (1.25,1.35) | 2.48 (2.38,2.58) |
| III | 11.43 (11.20,11.66) | 1.35 (1.24,1.46) | 1.13 (1.02,1.26) | 1.56 (1.48,1.64) | 1.87 (1.78,1.97) | 5.49 (5.27,5.72) |
| IV | 38.61 (38.08,39.14) | 1.85 (1.70,2.01) | 1.17 (1.04,1.33) | 2.50 (2.38,2.64) | 3.23 (3.08,3.40) | 12.94 (12.49,13.40) |
| Not determined | 10.77 (10.60,10.95) | 1.66 (1.57,1.76) | 1.15 (1.05,1.25) | 1.77 (1.70,1.84) | 2.28 (2.19,2.38) | 4.67 (4.50,4.85) |
|  |  |  |  |  |  |  |

AMI acute myocardial infarction, CI confidence interval, CV cardiovascular, HR hazards ratio, TIA transient ischemic attack

Adjusted for baseline age, biological sex, neighbourhood material deprivation quintile, rural/urban, distances to cancer centre, and distance to family doctor, plus 31 comorbidities: alcohol misuse, asthma, atrial fibrillation, heart failure, severe chronic kidney disease, chronic pain, chronic pulmonary disease, dyslipidemia, viral hepatitis B, cirrhosis, severe constipation, dementia, depression, diabetes mellitus, epilepsy, gout, hypertension, hypothyroidism, inflammatory bowel disease, irritable bowel syndrome, myocardial infarction, multiple sclerosis, severe obesity, osteoporosis, Parkinson’s disease, peptic ulcer disease, peripheral artery disease, psoriasis, rheumatoid arthritis, schizophrenia, and stroke/TIA.

**Supplemental Table 6. Baseline characteristics by cancer status during follow-up in 1:1 age-sex matches**

| **Characteristic** | **Cancer** | **No Cancer** |
| --- | --- | --- |
| N | 224,016 | 224,016 |
| Age, y | 56 [43,67] | 56 [43,67] |
| Female | 56.8 | 56.8 |
| Material deprivation quintile | 3 [2,4] | 3 [2,4] |
| Rural dwelling | 12.1 | 11.4 |
| Distance to health care, km |  |  |
| Cancer centre | 25 [10,55] | 25 [10,50] |
| Family doctor | 5 [5,5] | 5 [5,5] |
| *Cardiovascular* *comorbidities* |  |  |
| Dyslipidemia | 24.1 | 24.9 |
| Hypertension | 31.7 | 28.7 |
| Severe obesity | 17.1 | 12.7 |
| Diabetes | 10.1 | 9.5 |
| Prior stroke or transient ischemic attack | 4.6 | 4.7 |
| Heart failure | 3.2 | 3.5 |
| Atrial fibrillation | 3.0 | 3.1 |
| Myocardial infarction | 2.1 | 2.0 |
| Peripheral artery disease | 1.0 | 0.8 |
| *Non-cardiovascular comorbidities* |  |  |
| Chronic pain | 15.1 | 12.9 |
| Depression | 7.8 | 7.0 |
| COPD | 10.1 | 8.6 |
| Hypothyroidism | 7.3 | 7.0 |
| Osteoporosis | 6.9 | 6.7 |
| Gout | 5.8 | 5.1 |
| Alcohol misuse | 2.3 | 1.7 |
| Asthma | 2.1 | 2.0 |
| Irritable bowel syndrome | 1.5 | 1.4 |
| Epilepsy | 1.2 | 1.1 |
| Rheumatoid arthritis | 1.8 | 1.6 |
| Dementia | 0.7 | 1.6 |
| Schizophrenia | 0.7 | 0.7 |
| Inflammatory bowel disease | 0.8 | 0.7 |
| Multiple sclerosis | 0.7 | 0.7 |
| Severe constipation | 0.5 | 0.5 |
| Psoriasis | 0.6 | 0.5 |
| Severe chronic kidney disease | 0.4 | 0.6 |
| Parkinson’s disease | 0.4 | 0.4 |
| Peptic ulcer disease | 0.2 | 0.2 |
| Cirrhosis | 0.2 | 0.1 |
| Chronic hepatitis B | 0.1 | 0.1 |
|  |  |  |

COPD chronic obstructive pulmonary disease

All characteristics were obtained at baseline. Participants in the cancer group developed cancer at some point during follow-up. All measures are expressed as percentages or medians [Q1,Q2]. The following variables are missing data: material deprivation quintile (8.6%), rural dwelling (6.7%), distance to cancer centre (7.9%), family doctor (6.9%), severe obesity (11.5%) and dyslipidemia (14.8%).

**Supplemental Table 7. Cardiovascular risk by cancer status within age groups**

| **Cancer stage** | **All-cause mortality** | **CV mortality** | **AMI** | **Stroke** | **Heart**  **failure** | **Pulmonary**  **embolism** |
| --- | --- | --- | --- | --- | --- | --- |
|  | **HR (95% CI)** | **HR (95% CI)** | **HR (95% CI)** | **HR (95% CI)** | **HR (95% CI)** | **HR (95% CI)** |
| Cancer vs no cancer |  |  |  |  |  |  |
| 18-49y | 22.83 (22.20,23.48) | 1.75 (1.45,2.10) | 1.07 (0.94,1.21) | 1.93 (1.83,2.03) | 2.42 (2.26,2.59) | 3.94 (3.80,4.08) |
| 50-59y | 19.83 (19.34,20.33) | 1.58 (1.43,1.74) | 0.91 (0.84,0.99) | 1.58 (1.51,1.65) | 1.78 (1.69,1.87) | 4.50 (4.34,4.67) |
| 60-69y | 11.38 (11.14,11.63) | 1.42 (1.33,1.52) | 1.04 (0.97,1.12) | 1.36 (1.31,1.41) | 1.57 (1.51,1.63) | 3.37 (3.25,3.50) |
| 70-79y | 6.08 (5.97,6.20) | 1.32 (1.26,1.38) | 1.11 (1.04,1.20) | 1.26 (1.22,1.31) | 1.46 (1.41,1.52) | 2.57 (2.47,2.68) |
| ≥80y | 3.88 (3.80,3.97) | 1.16 (1.10,1.22) | 1.05 (0.93,1.17) | 1.27 (1.20,1.34) | 1.45 (1.39,1.53) | 2.02 (1.87,2.18) |
|  |  |  |  |  |  |  |

AMI acute myocardial infarction, CI confidence interval, CV cardiovascular, HR hazards ratio, TIA transient ischemic attack

Adjusted for baseline age, biological sex, neighbourhood material deprivation quintile, rural/urban, distances to cancer centre, and distance to family doctor, plus 31 comorbidities: alcohol misuse, asthma, atrial fibrillation, heart failure, severe chronic kidney disease, chronic pain, chronic pulmonary disease, dyslipidemia, viral hepatitis B, cirrhosis, severe constipation, dementia, depression, diabetes mellitus, epilepsy, gout, hypertension, hypothyroidism, inflammatory bowel disease, irritable bowel syndrome, myocardial infarction, multiple sclerosis, severe obesity, osteoporosis, Parkinson’s disease, peptic ulcer disease, peripheral artery disease, psoriasis, rheumatoid arthritis, schizophrenia, and stroke/TIA.
